# RANK/RANK-L/OPG in Patients with Bone Metastases Treated with Anticancer Agents and Zoledronic Acid: A Prospective Study

**DOI:** 10.3390/ijms140610683

**Published:** 2013-05-23

**Authors:** Laura Mercatali, Marianna Ricci, Emanuela Scarpi, Patrizia Serra, Francesca Fabbri, Rossana Ricci, Chiara Liverani, Michele Zanoni, Wainer Zoli, Roberta Maltoni, Erica Gunelli, Dino Amadori, Toni Ibrahim

**Affiliations:** 1 Osteoncology and Rare Tumors Center, IRCCS Istituto Scientifico Romagnolo per lo Studio e la Cura dei Tumori (IRST), via P. Maroncelli 40, 47014 Meldola (FC), Italy; E-Mails: l.mercatali@irst.emr.it (L.M.); marianna.ricci@irst.emr.it (M.R.); rossana.ricci@ausl.fo.it (R.R.); c.liverani@irst.emr.it (C.L.); ee219@libero.it (E.G.); d.amadori@irst.emr.it (D.A.); 2 Biosciences Laboratory, IRCCS Istituto Scientifico Romagnolo per lo Studio e la Cura dei Tumori (IRST), via P. Maroncelli 40, 47014 Meldola (FC), Italy; E-Mails: m.zanoni@irst.emr.it (M.Z.); w.zoli@irst.emr.it (W.Z.); 3 Unit of Biostatistics and Clinical Trials, IRCCS Istituto Scientifico Romagnolo per lo Studio e la Cura dei Tumori (IRST), via P. Maroncelli 40, 47014 Meldola (FC), Italy; E-Mail: e.scarpi@irst.emr.it (E.S.); p.serra@irst.emr.it (P.S.); francesca.fabbri@irst.emr.it (F.F.); 4 Department of Medical Oncology, IRCCS Istituto Scientifico Romagnolo per lo Studio e la Cura dei Tumori (IRST), via P. Maroncelli 40, 47014 Meldola (FC), Italy; E-Mail: r.maltoni@irst.emr.it

**Keywords:** RANK, RANK-L, OPG, NTX, bone metastases

## Abstract

Patients with solid cancer frequently develop bone metastases (BM). Zoledronic acid (Zometa^®^, ZA), routinely used to treat patients with BM, acts on osteoclasts and also has antitumor properties. We aimed to assess the effect of ZA over time in novel bone turnover markers (RANK/receptor activator of nuclear factor-k B ligand (RANK-L)/Osteoprotegerin (OPG)) and to correlate these with serum N-terminal telopeptide (NTX). The study prospectively evaluated levels of RANK, RANK-L and OPG transcripts by real-time PCR and NTX expression by ELISA in the peripheral blood of 49 consecutive patients with advanced breast, lung or prostate cancer. All patients received the standard ZA schedule and were monitored for 12 months. Median baseline values of RANK, RANK-L and OPG were 78.28 (range 7.34–620.64), 319.06 (21.42–1884.41) and 1.52 (0.10–58.02), respectively. At 12 months, the median RANK-L value had decreased by 22% with respect to the baseline, whereas median OPG levels had increased by about 96%. Consequently, the RANK-L/OPG ratio decreased by 56% from the baseline. Median serum NTX levels decreased over the 12-month period, reaching statistical significance (*p* < 0.0001). Our results would seem to indicate that ZA modulates RANK, RANK-L and OPG expression, thus decreasing osteoclast activity.

## 1. Introduction

Bone metastases are common in many solid tumors, in particular breast, prostate and lung cancer. In the United States, about two-thirds of patients who die from cancer each year have metastatic bone disease [1,2]. Approximately 20%–25% of patients with cancer develop clinically evident bone metastases, and a further 50% of these have lesions identified at autopsy [3,4]. Metastatic bone disease disrupts normal bone homeostasis, a dynamic process involving osteoclast-mediated osteolysis and osteoblast-mediated osteogenesis. Increasingly unbalanced bone metabolism leads to a loss of bone integrity, which may result in skeletal-related events (SREs), such as bone pain, pathological fractures, spinal cord compression or hypercalcemia. The frequency of SREs in these patients depends mainly on the site of metastasis, type of lesion (lytic or blastic) and previous treatment received [5].

Bisphosphonates play a key role in the treatment of bone metastases [6], positively influencing the natural history of the disease, improving quality of life and decreasing pain and the frequency of skeletal-related events. The most widely used bisphosphonate is zoledronic acid (ZA) [6–9]. Several circulating markers have been studied for their potential to predict response to ZA in patients with bone metastases, without, however, encouraging results. Urinary *n*-telopeptide (NTX) is the marker most frequently analyzed in studies of large case series [10–15], and normalization of urinary NTX values after three months’ treatment with ZA has been associated, in retrospective studies, with reduced risks of both skeletal complications and death [16].

The molecules involved in the RANK/RANK-L/OPG axis, which governs osteoclastogenesis and bone resorption, are also candidate markers [17,18]. The receptor activator of nuclear factor-k B ligand (RANK-L) binds and activates its receptor, RANK, on the surface of osteoclasts, stimulating osteoclast differentiation and maturation, inhibiting osteoclast apoptosis and increasing bone resorption. Osteoprotegerin (OPG), a member of the TNF receptor family, is secreted by various cell types, including osteogenic cells, and acts as a decoy receptor of RANK-L, thereby inhibiting osteoclastogenesis. It has been hypothesized that the RANK/RANK-L/OPG pathway may be modulated by ZA, because of its involvement in the modulation of bone resorption.

Circulating bone turnover markers of resorption and formation have been evaluated for their ability to act as indicators of activity of bisphosphonates in metastatic bone disease.

The aim of this prospective study was to evaluate the effect of ZA over time on novel circulating bone turnover markers (RANK/RANK-L/OPG) and to correlate these with serum NTX.

## 2. Results

Patient characteristics are listed in Table 1. Biological and pathological data on breast cancer patients are reported on Table 2, and clinical data on bone lesions and other sites of distant metastases are shown in Table 3.

### 2.1. RANK/RANK-L/OPG Pathways

**Figure 1:**
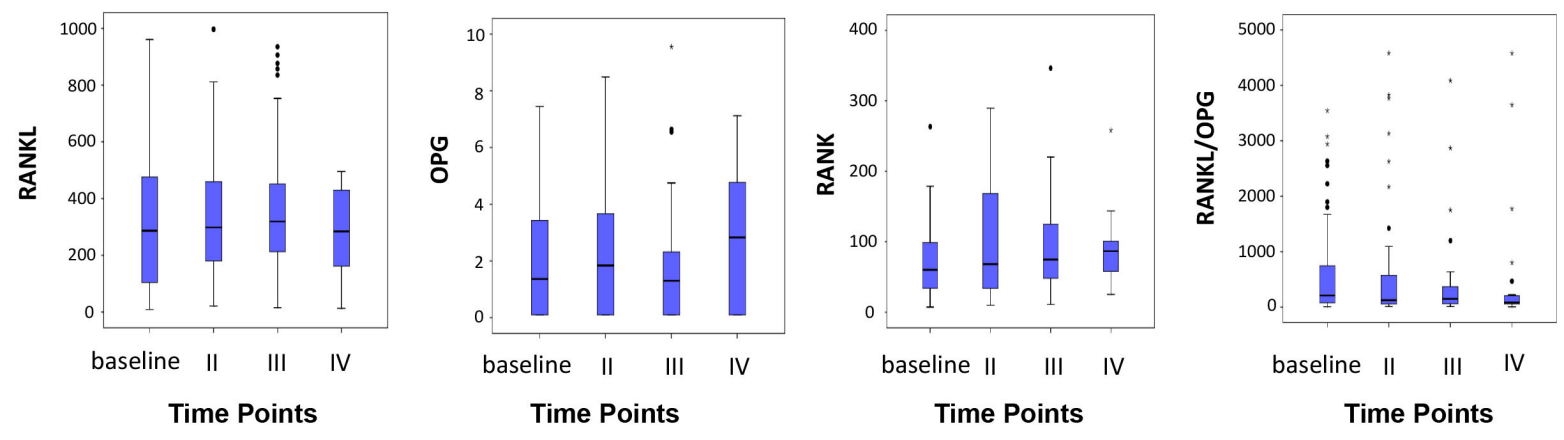
Marker variations over time.

Considering the entire case series, median baseline RANK, RANK-L and OPG values were 78.28 (range 7.34–620.64), 319.06 (21.42–1884.41) and 1.52 (0.10–58.02), respectively. At 9–12 months, median RANK-L values had decreased by 22% with respect to the baseline, while median OPG levels had increased by about 96%. Consequently, the RANK-L/OPG ratio decreased by 56% from the baseline. These differences, however, did not reach statistical significance. Median RANK values had risen by 36% at 6–8 months, but showed a return towards baseline values at 9–12 months (*p* = 0.013) (Figure 1). OPG, RANK and RANK-L/OPG ratio levels were significantly related.

### 2.2. NTX

**Figure 2:**
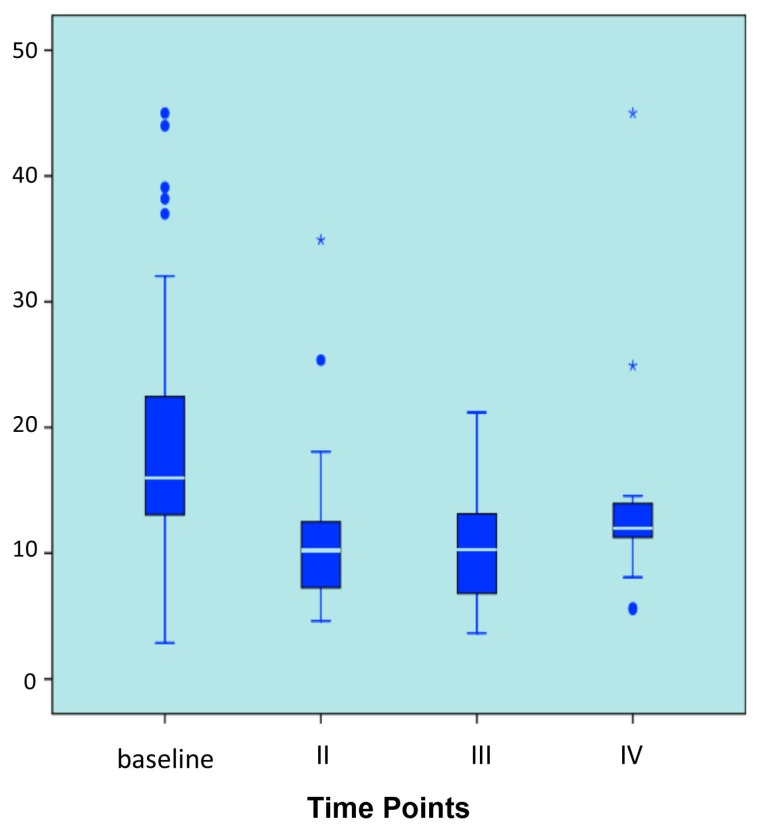
N-terminal telopeptide (NTX) variation over time.

The median baseline NTX value was 15.65 nm bone collagen equivalent BCE (range 2.85–45.00). Median values were 10.80 nm BCE (4.62–34.91) at 3–4 months, 9.99 nm BCE (3.64–21.2) at 6–8 months and 10.91 nm BCE (5.53–45.00) at 9–12 months. Median serum NTX levels at 3–4 and at 6–8 months were 35% and 39% lower than those of baseline, respectively, reaching statistical significance (*p* < 0.0001). The median NTX value at 9–12 months showed a 26% decrease with respect to the baseline (Figure 2).

### 2.3. Breast Cancer Patients

#### CEA and CA15-3

The median baseline CEA value of 7.32 ng/mL (range 25.12–1264.09) had decreased to 4.10 (1.00–1122.10) at 9–12 months (not statistically significant). CA15-3 showed a median baseline value of 40.00 ng/mL (range 1.50–809.00), increasing to 48.60 (6.20–1633.00) at 9–12 months (not statistically significant). No correlation was found between CEA and CA15-3 and the other markers evaluated.

## 3. Discussion

In a previous case control study, we observed that median OPG levels of bone metastases were significantly lower in BM patients compared to disease-free individuals or healthy donors [19]. Furthermore, no significant differences were seen in RANK-L and OPG levels as a function of age and menopausal status. Taking into consideration the role played by zoledronic acid in the inhibition of bone turnover, we decided to evaluate the RANK/RANK-L/OPG pathway to understand whether ZA also exerts its effect on bone resorption through this pathway. To our knowledge, this is one of the few prospective studies to assess circulating marker changes in response to ZA in patients with bone metastases from solid tumors. We evaluated about fifty patients, 73% of whom had breast cancer as the primary tumor. Results were interesting because, after 12 months of treatment (12 ZA infusions), we observed a decrease in RANK-L in the overall case series, accompanied by an increase in OPG, with a consequent decrease in the RANK-L/OPG ratio. This last event has important biological implications, suggesting that ZA over time may influence the expression pattern of molecules involved in pre-osteoclast activation. The RANK trend over time was less linear than that of RANK-L or OPG, showing an increase at 6–8 months and a return towards baseline values at 9–12 months. Such heterogeneity could be explained by the fact that RANK-L and OPG are produced mainly by bone and immune system cells, whereas RANK expression may depend on cancer cell expression [20,21]. We only obtained statistically significant results for NTX [22–26]. In a similarly sized case series, Mountzios and coworkers observed a decrease in the RANK-L/OPG ratio, albeit not statistically significant, following a reduction in only RANK-L values. It must be pointed out here that we measured mRNA extracted from whole blood, whereas Mountzios evaluated protein concentrations from serum samples, stating that results should be interpreted with caution, because RANK-L measures were invalidated by the limited stability of the serum isoform.

Our evaluation of NTX, widely used to monitor metastatic bone disease, confirmed previous observations of a significant decrease in the marker after ZA treatment. Interestingly, this decrease was already present at the first follow-up evaluation. NTX was not related to other markers and showed different behavior when analyzed by subgroup. The RANK-L/OPG ratio was found to be a prognostic factor for survival in multiple myeloma [27].

CEA and CA15-3, markers used in routine clinical practice for breast cancer patients, showed no correlation with other markers. Zhao and coworkers reported a normalization of CEA and CA15-3 values in patients treated with weekly ZA rather than the standard monthly schedule [22]. We did not monitor the role of CEA and PSA in lung and prostate cancer, respectively, because of the small number of patients in our study. Our results would seem to indicate that markers of the RANK/RANK-L/OPG pathway could be better at identifying neoplastic bone involvement than conventional tumor markers.

## 4. Materials and Methods

### 4.1. Study Design

This prospective study was carried out at IRCCS Istituto Scientifico Romagnolo per lo Studio e la Cura dei Tumori in Meldola, Italy. Our primary objective was to evaluate the trend of circulating markers over time. Chosen markers were OPG, RANK-L and RANK transcripts and serum NTX protein levels. We also aimed to correlate results with those obtained using conventional tumor markers, carcinoembryonic antigen (CEA) and CA15-3. Furthermore, all markers were correlated with biological parameters of the primary tumor. The protocol was reviewed and approved by the local ethics committee and performed according to Good Clinical Practice and the Helsinki declaration. The patients gave their written informed consent to take part in the study.

### 4.2. Case Series

Peripheral venous blood (PB) samples were obtained from 49 consecutive patients with bone metastases from solid tumors consecutively enrolled from March 2007 to December 2009. Thirty-six patients had breast cancer, 7 prostate cancer and 6 lung cancer. Eligibility criteria were as follows: males/females age ≥18 years; histological confirmation of solid cancer; radiological confirmation of bone metastases; performance status ≤2; life expectancy >6 months; normal renal and hepatic function with total bilirubin <2 mg/dL; and serum creatinine <2 mg/dL. Exclusion criteria comprised: history of postmenopausal osteoporosis; previous treatment with bisphosphonates, presence of osteonecrosis of the jaw and dental conditions requiring oral cavity surgery. None of the patients had active cardiac disease.

### 4.3. Treatment, Blood Collection, Follow Up

Patients received a 15 min intravenous infusion of ZA 4 mg (Zometa, Novartis, East Hanover, NJ, USA) in 100 mL of 0.9% saline every 28 days. Follow up consisted of blood tests and instrumental radiological exams every 3–4 months after the diagnosis of bone metastases for about one year.

The first blood sample was collected at the moment of the bone metastasis diagnosis, before any specific treatment.

### 4.4. Biological Samples

PB samples were collected at each re-evaluation, left to coagulate for 30 min at room temperature and centrifuged at 2,000× *g* for 15 min, after which serum was stored at −80 °C until assays were performed. Pax-gene tubes were left at least two hours at room temperature, stored overnight at −20 °C and then at −80 °C until RNA extraction was performed.

### 4.5. RANK/RANK-L/OPG

Blood RNA was extracted by PAX-Gene blood RNA kit (PreAnalytix-Qiagen, Hilden, Germany) according to the manufacturer’s instructions. RNA was treated with DNAse I (Qiagen, Hilden, Germany), and 500 ng of RNA were reverse-transcribed using the iScript cDNA Synthesis Kit (BioRad, Hercules, CA, USA). Real-time PCR was performed using the MyiQ Single Color Real-Time PCR Detection System (BioRad, Hercules, CA, USA) and SYBR Green I dye chemistry. The stably expressed endogenous, β*-actin* and *HPRT* genes, were amplified and used as reference genes. Primers were designed by Beacon Designer Software (Premier Biosoft International, Palo Alto, CA, USA). Primer sequences and the real-time PCR thermic profile have been reported previously [28]. The type of analysis is a relative expression of the transcript with respect to the mRNA of a healthy donor: for this reason, the expression of RANK/RANK-L/OPG do not need a unit.

### 4.6. NTX Immunoassay

NTX levels were measured by a competitive-inhibition enzyme-linked immunosorbent assay (ELISA/EIA) (Osteomark, Princeton, NJ, USA). The assays were performed following the manufacturer’s instructions. All samples were tested in duplicate for both markers. For both analyses, all samples from the same individuals were analyzed on the same experimental plate. Immunoassays were performed according to the manufacturers’ instructions.

With regard to intra-assay reproducibility, all determinations were performed in duplicate and repeated when the coefficient of variation (CV) exceeded 10%. An internal control was added in all assays to assess inter-assay reproducibility. Inter-assay CV was always less than 15%.

### 4.7. Carcinoembryonic Antigen and CA15-3

CEA and CA 15-3 assays were routinely performed in the Clinical Pathology Laboratory of Morgagni-Pierantoni Hospital in Forlì using AxSYM Chemiluminescent Microparticle Immunoassay (CMIA) and Architect Microparticle Enzyme Immunoassay (MEIA; Abbott Laboratories, Abbott Park, III, USA), respectively. The markers were only used to evaluate breast cancer patients.

### 4.8. Statistical Analysis

The median value of each marker, considered as a continuous variable, was calculated at the time of the diagnosis of bone involvement (“baseline” measurement) and every 3–4 months thereafter.

A comparison of marker values over time (observations repeated in the same subjects) was made using nonparametric ranking statistics (Friedman test). Another nonparametric ranking statistic method, the median test, was used to analyze the relationship between serum levels of each marker and patient characteristics. Spearman’s rank correlation test was used to investigate the relation between baseline values of each marker. Statistical analyses were carried out with SAS Statistical software (version 9.1, SAS Institute, Cary, NC, USA).

## 5. Conclusions

In conclusion, the present prospective study highlights a clear trend towards a decrease in the RANK-L/OPG ratio and NTX levels after 12 months of treatment with zoledronic acid. Our findings concerning the decrease in the RANK-L/OPG ratio raises the interesting hypothesis that this drug may also influence osteoclast behavior during differentiation, perhaps via an indirect mechanism that leads to a block in the vicious cycle of bone metastases. Further investigations into this area are warranted.

## Figures and Tables

**Table 1:** Patient characteristics: overall case series.

	Cancer patients			

**Features**	Total	BC *	PCa §	Lung
	No. (%)	No. (%)	No. (%)	No. (%)
**Overall**	49	36	7	6
Median age, years (range)	62 (34–86)	59 (34–86)	70 (51–83)	60 (47–68)
Sex: males	12 (24.5)	0	7 (100)	5 (83.3)
females	37 (75.5)	36 (100)	0	1 (16.7)
**Performance status**				
0	40 (81.6)	30 (83.3)	6 (85.7)	4 (66.7)
1	8 (16.3)	6 (16.7)	0	2 (33.3)
2	1 (2.1)	0	1 (14.3)	0

Note:

* BC, breast cancer;

§ PCa, prostate cancer.

**Table 2:** Bio-pathological features of BC primary tumors.

Features	No. (%)
**Histology**	
Ductal	21 (58.3)
Lobular	8 (22.3)
Adenocarcinoma	7 (19.4)
**Grading**	
1	2(5.5)
2	14 (38.9)
3	20 (55.6)
ER*	
<10%	3 (8.3)
≥10%	33 (91.7)
**PgR**§	
<10%	12 (33.3)
≥10%	24 (66.7)
**Ki67**	
Low	12 (33.3)
High	24 (66.7)
**HER2**	
Not amplified	23 (63.9)
Amplified	13 (36.1)
**Menopausal status**	
Premenopausal	6 (16.7)
Perimenopausal	2 (5.5)
Postmenopausal	28 (77.8)
**Therapies**	
Neoadjuvant	3 (9.4)
Adjuvant	28( 82.4)
Chemotherapy	24 (75.0)
Hormone	26 (81.2)
Biological	3 (9.7)
**Lines of therapy**	
1	16 (44.5)
2	13 (36.1)
3	7 (19.4)

Note:

* ER, estrogen receptor;

§ PgR, progesterone receptor.

**Table 3:** Metastasis features of cancer patients. BM, bone metastases. SREs, skeletal-related events.

	Cancer Patients			
	
	Total (%)	BC (%)	PCa (%)	Lung (%)
Visceral metastases				
Presence	25 (51.0)	15 (41.7)	5 (71.4)	5 (83.3)
Absence	24 (49.0)	21 (58.3)	2 (28.6)	1 (16.7)

Bone lesion type				
Osteolytic	24 (55.8)	20 (58.8)	1 (25.0)	3 (60.0)
Osteoblastic	13 (30.2)	10 (29.4)	3 (75.0)	0
Mixed	6 (14.0)	4 (11.8)	0	2 (40.0)
Missing	6	2	3	1

No. of BM sites				
1	5 (10.2)	4 (11.1)	1 (14.3)	0
2–6	34 (69.4)	25 (69.4)	4 (57.1)	5 (83.3)
>6	10 (20.4)	7 (19.4)	2 (28.6)	1 (16.7)

No. of SREs	7	6 (85.7)	1 (14.3)	0
